# Uncovering the Burden of Dengue in Africa: Considerations on Magnitude, Misdiagnosis, and Ancestry

**DOI:** 10.3390/v14020233

**Published:** 2022-01-25

**Authors:** Emily Mary Gainor, Eva Harris, A. Desiree LaBeaud

**Affiliations:** 1 Division of Infectious Diseases and Vaccinology, School of Public Health, University of California, Berkeley, CA 94720-3370, USA; eharris@berkeley.edu; 2 Department of Pediatrics, Division of Infectious Diseases, Stanford University School of Medicine, Stanford, CA 94305, USA; dlabeaud@stanford.edu

**Keywords:** dengue, Africa, *Aedes*, epidemiology, climate change, outbreaks, mosquitoes, misdiagnosis, coinfection, ancestry

## Abstract

Dengue is a re-emerging neglected disease of major public health importance. This review highlights important considerations for dengue disease in Africa, including epidemiology and underestimation of disease burden in African countries, issues with malaria misdiagnosis and co-infections, and potential evidence of genetic protection from severe dengue disease in populations of African descent. The findings indicate that dengue virus prevalence in African countries and populations may be more widespread than reported data suggests, and that the *Aedes* mosquito vectors appear to be increasing in dissemination and number. Changes in climate, population, and plastic pollution are expected to worsen the dengue situation in Africa. Dengue misdiagnosis is also a problem in Africa, especially due to the typical non-specific clinical presentation of dengue leading to misdiagnosis as malaria. Finally, research suggests that a protective genetic component against severe dengue exists in African descent populations, but further studies should be conducted to strengthen this association in various populations, taking into consideration socioeconomic factors that may contribute to these findings. The main takeaway is that Africa should not be overlooked when it comes to dengue, and more attention and resources should be devoted to this disease in Africa.

## 1. Introduction

Dengue is a re-emerging and neglected infectious disease of great public health importance [1]. The United States Center for Disease Control and Prevention (CDC) estimates that forty percent of the globe’s inhabitants live in areas where dengue acquisition is a risk, and up to 400 million people are infected with dengue virus (DENV) every year, 50–100 million people get sick, and 20,000 people die from severe dengue [2,3]. Dengue cases in Africa have been documented since as early as 1779, but there has been fragmented reporting of the epidemiology of this disease and a lack of a comprehensive review concerning the various considerations relevant to this underreported and overlooked problem on the African continent [4]. In Africa specifically, estimates from 2010 suggest that there were 15.7 million apparent DENV infections (any disease severity level), and 48.4 million inapparent infections during the year [5]. Modeling further suggests that the burden of dengue in Africa and the Americas is roughly equivalent, despite the Americas historically receiving significantly more attention when it comes to dengue burden compared to Africa [5]. Dengue also exists in sylvatic cycles in African forests between mosquitoes and non-human primates, and while spillover can occur, it is not considered to be common [6]. Besides humans in Africa, DENV has been observed to infect nonhuman primates (particularly monkeys), domestic mammals, birds, and even a buffalo [6].

Dengue virus belongs to the *Flaviviridae* family, Flavivirus genus, and is comprised of four distinct serotypes (DENV-1, DENV-2, DENV-3, and DENV-4) [7,8]. Dengue is an arboviral disease, and the virus is spread predominantly by two mosquito vectors, *Aedes aegypti* and *Aedes albopictus* [9]. Acquiring any one of these serotypes will typically confer lifelong immunity to disease caused by that serotype, but not necessarily protection against the other serotypes, so individuals may be affected up to four times over their lifetime [10]. DENV has the potential to cause a range of disease symptoms, from asymptomatic infection (50–75% of infections), to mild, to severe, and even deadly manifestations. Dengue disease has traditionally been categorized (according to the 1997 WHO guidelines) as undifferentiated febrile illness (a mild form with fever), dengue fever (incapacitating, but death is unlikely), dengue hemorrhagic fever (DHF; involving plasma leakage and low platelet count, or thrombocytopenia), and dengue shock syndrome (DSS; DHF where the leakage of plasma leads to hypotension and shock) [11,12]. As of 2009, the new WHO disease classifications are “Dengue with or without Warning Signs” and “Severe Dengue” [13]. While DHF/DSS only accounts for around 1–2% of dengue, this is still a significant burden given the large percentage of the world’s population that is affected by DENV annually and the lack of therapies or an effective vaccine [10,12]. Non-severe dengue manifests as a nonspecific febrile illness; thus, global dengue burden may be further underestimated, a problem compounded by a lack of surveillance systems in lower-resource countries [14]. Dengue has become endemic in Asia, Africa, Latin America and the Caribbean, and the Pacific [15]. In Africa, other medically important flaviviruses, such as West Nile virus, Japanese encephalitis virus, yellow fever virus, and Zika virus may co-circulate in the same overlapping geographic areas, as well as a variety of lesser-known flaviviruses including Spondweni virus, Wesselsbron virus, and Bagaza Virus [16,17,18]. These viruses and their clinical manifestations complicate the estimation of dengue burden.

New problems continue to emerge that complicate the public health response to dengue. There is substantial scientific evidence supporting an increased risk of severe disease in secondary DENV infections [19,20]. Different sequences of infection based on the serotype confer different levels of risk for development of severe dengue, and some studies suggest a higher risk for disease severity with a secondary infection of DENV-2 [19,21]. The proposed mechanism for the increased severity risk is antibody-dependent enhancement (ADE). ADE not only increases the risk of severe dengue in secondary infections, but it also complicates the development of a vaccine for dengue. Dengvaxia^®^, developed by Sanofi Pasteur, is the only licensed dengue vaccine, and it cannot be used in dengue-seronegative patients, since it can increase the risk of hospitalization and severe dengue in seronegative vaccinees upon subsequent natural DENV infection [22]. Therefore, this is not a viable option for those living in dengue-endemic areas who are seronegative (e.g., children) nor for those traveling to dengue-endemic areas. Zika virus also complicates the use of Dengvaxia^®^ use due to antigenic cross-reactivity with DENV [23].

Climate change is another pertinent concern. Dengue is a climate-sensitive disease, and models show that climate can predict mosquito-borne disease dynamics in Africa [24]. As the Earth continues to warm over time, vectors that spread important arboviral diseases, including dengue, can better proliferate and spread into more temperate zones. The extrinsic incubation period, or the time that DENV remains in the mosquito after a blood meal before it disseminates to the salivary glands and can be transmitted to other humans, is shorter in warm climates [25]. Increases in water temperature also result in more rapid maturation of mosquito larvae, and warmer climates tend to correlate with an increased number of blood meals by female mosquitoes, leaving more opportunities for transmission of viruses [26]. Research also suggests that abnormally wet months are later associated with an abundance of mosquito eggs and adults [27]. Additionally, as urbanization continues to rise, global travel and trade increases, and waste such as plastic containers and tires build up, *Aedes* vector competence and spread will increase [28]. In urban environments, there is a documented association between *Aedes*-borne diseases such as dengue and solid-waste accumulation [29]. Dengue outbreaks are becoming increasingly common in rural environments globally as well [30].

Dengue is a topic of global public health importance, especially in low-resource countries [31]. Dengue spread on the African continent is an emerging problem. Many sub-Saharan African countries are severely resource-constrained and therefore may face issues with proper diagnostic testing, vector control, and medical attention to dengue [32,33]. Not only is dengue medically important and a major cause of morbidity worldwide, but it is also associated with significant economic burden, in the magnitude of billions of dollars annually [34]. This review will focus on the burden of dengue in African populations. Research into this topic revealed three major themes particularly relevant for DENV in Africa. One theme is the general epidemiology of dengue in Africa and how it has changed over time, including geographical dissemination of disease and the degree to which dengue burden in Africa is underestimated. A second theme, related to this underestimation, is the relationship between diffuse malaria prevalence in Africa and dengue, including discussions on misdiagnosis of these febrile illnesses as well as the burden of co-infection with both agents. Finally, this review will discuss a central question of debate: whether people of African descent are protected from severe dengue compared to other ancestral groups.

## 2. Methods

**Figure 1:**
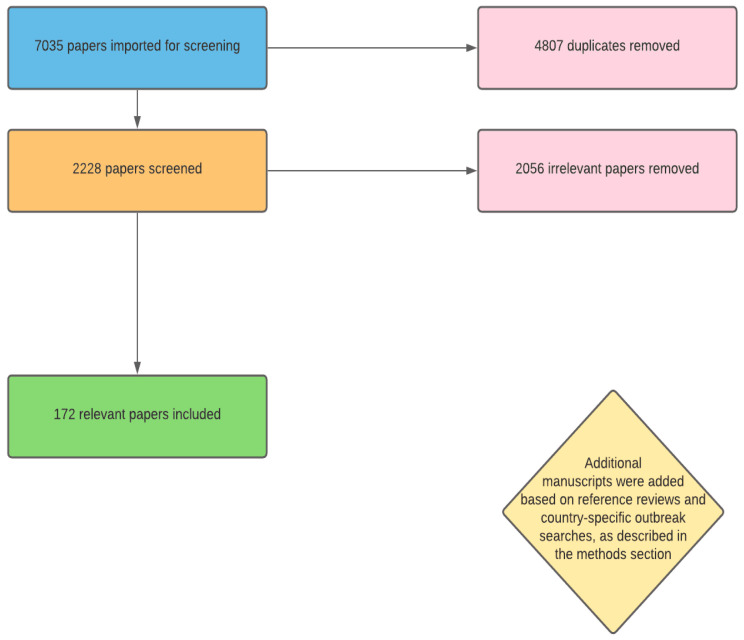
PRISMA-like diagram outlining the methods for article screening.

An electronic literature review was conducted using the PubMed and Embase databases. The search terms used in both databases were “Dengue Africa”, “Dengue Africa epidemiology”, “Dengue fever Africa”, “Dengue malaria misdiagnosis”, “Dengue malaria misclassified”, “Dengue malaria coinfection”, “Dengue malaria Africa”, “Dengue severity African descent”, “Dengue severity Black patients”, “Dengue hemorrhagic fever Black patients”, and “Dengue shock syndrome Black patients”. These searches yielded a total of 7035 studies. The removal of 4807 duplicates left 2228 studies to be screened. Title and abstract screening using Covidence systematic review software (Veritas Health Innovation, Melbourne, Australia) removed 2056 irrelevant studies. Studies were deemed irrelevant if they did not focus on dengue in Africa, on dengue severity based on host ancestry or genetics, or on dengue and malaria misdiagnosis or *Plasmodium* spp./DENV co-infection. Additionally, studies focusing on transmission in non-human species were excluded, unless the papers were discussing the spread or prevalence of mosquito vectors in Africa that are known to spread DENV, as these were useful for analysis of future burden. This left a total of approximately 172 manuscripts to be examined for the purposes of this review. Some manuscripts included in this total count that were significantly similar in nature to others were subsequently excluded in order to avoid duplication of information as the review materialized. While this is a literature review and not a systematic review, Figure 1 is a PRISMA-like diagram outlining these methods, as similar methods were followed for manuscript identification. References of papers were also screened, and additional manuscripts relevant to this review were included. An additional search was undertaken for each independent African country (French, Spanish and other territories excluded) using the search term “[country name] dengue outbreaks” (Example: “Angola dengue outbreaks”).

## 3. Results and Discussion

### 3.1. Dengue in Africa: Epidemiologic Characteristics and Considerations

Dengue cases were first recorded in Africa in 1779 [4]. Since then, there have been a series of outbreaks across the continent of all four DENV serotypes, including in Kenya, Benin, Cape Verde, Angola, Tanzania, Somalia, and the Comoros [30,35,36,37]. In the region of the Middle East and North Africa (MENA), the few outbreaks recorded in the African portion of this area have been in South Sudan, with one additional outbreak in Egypt [38]. The rest of the outbreaks on the African continent have been recorded in sub-Saharan Africa. Generally, outbreaks of dengue in Africa are not very common based on reported data, especially severe dengue. However, this does not necessarily mean that dengue and severe dengue are rare in Africa. Underreporting and misdiagnosis will be discussed in a later section, along with how a lack of accurate identification may be downplaying the dengue situation in Africa.

Outbreaks have been sporadic over time and are caused by either distinct or co-circulating DENV serotypes [39]. All four serotypes are transmitted in Africa. DENV-2 has caused the most outbreaks compared to the other three serotypes, followed by DENV-1 [35,36]. Table 1 categorizes countries in the African continent based on their United Nations subregion classification [40]. Records of outbreaks were compiled from papers published within the last ten years (from January 2011 to June 2021) in order to provide a more up-to-date review of dengue activity. French, Spanish, and any other overseas territories or autonomous regions of the African continent and outlying islands such as Mayotte, the Canary Islands, and the French Southern Territories were excluded for the purpose of this analysis. Table 1 outlines the five subregion classifications (North Africa, East Africa, Central Africa, West Africa, and Southern Africa) and lists outbreaks that have been recorded by year for the past ten years. The United States CDC’s “Level of Risk” for dengue is also included [41]. It is important to note that most countries with no reported outbreaks in the last decade, barring the majority of North Africa, still had cases of dengue and may fall into the endemic category, especially if they have a listed CDC level of risk. Additionally, many of these countries have also had outbreaks in the past, outside of the span of the last decade, and therefore still have epidemic potential in the future [8]. Historically, outbreaks of dengue in Africa date back to 1823, and laboratory-confirmed outbreaks have been identified in over 20 African countries since then [37]. A series of papers already detail older outbreaks; therefore, this review focuses on the identification of more recent outbreaks. Additionally, due to limitations in laboratory testing and clinical diagnosis, not all dengue outbreaks are identified and reported; thus, the results of this table may be an underestimation of the true burden of dengue outbreaks in Africa. This problem will be discussed further below.

The results show that dengue outbreaks in the past decade (2011–2021) in Africa have occurred in a number of countries (16), and the subregion with the highest number of recent outbreaks is East Africa, followed by West Africa, North Africa, and then Central Africa. This ranking is interesting, as various reviews and seroprevalence studies have produced conflicting results. A review study on dengue in Africa similarly reported recent outbreaks to be higher in Eastern Africa, as well as studies that have found up to 30–50% seroprevalence in East African countries including Sudan and Kenya [30,70,71]. In contrast, a systematic review/meta-analysis of dengue prevalence studies in Africa found that IgG seroprevalence for dengue was 3.6% in Eastern Africa and 52.6% in Western Africa in the years 2000 to 2019, including studies that tested both healthy and febrile patients [37]. No outbreaks were reported/confirmed in Southern Africa in this decade. Some countries bordering countries experiencing an outbreak, such as those near Angola, Tanzania, Burkina Faso, Kenya, and Ethiopia also experienced increased cases of dengue and death attributed to dengue from citizens who crossed the borders into these countries during these outbreaks, as described in articles cited in Table 1.

**Figure 2:**
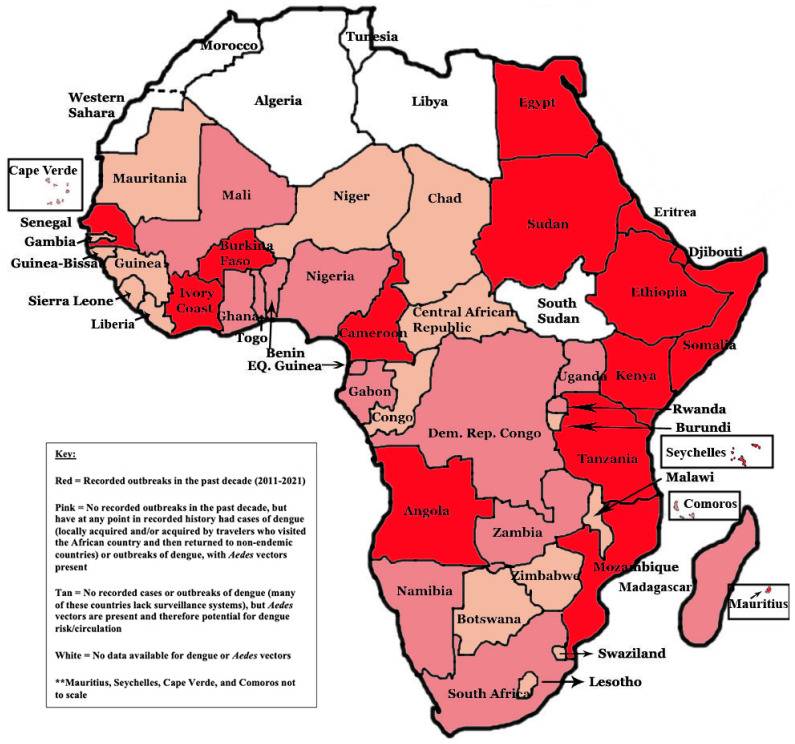
Mapping the epidemiology of dengue in Africa [30,35]; see Table 1 references. Notes: In 2011, the Republic of South Sudan seceded from Sudan. Therefore, it is difficult to determine whether dengue and/or *Aedes* exist in both Sudan and South Sudan due to the recent split and a lack of updated data. Thus, while South Sudan is coded as “no data available” and remains colored white for this map, it is likely that in the past, when the two countries were still combined, dengue cases and/or transmission had occurred in what is now considered South Sudan based on geographic proximity to other dengue-endemic countries and environmental suitability. These results are based on recorded cases only. Not every country has surveillance systems in place for dengue, but transmission may be possible based on vector competence and geography, and transmission may be occurring unrecognizably.

In addition to recorded outbreaks, there is evidence that dengue is endemic in at least 34 African countries [35]. This evidence is based on laboratory-reported cases, seroprevalence studies, and clinically suspected cases without laboratory confirmation [30,35,37]. Severe dengue is not very common in Africa. From 2011–2019, there were 176 severe cases of dengue, and most of these were co-infections with malaria [72]. It is difficult to find Africa-specific data on mortality rates and clinical outcomes in the literature. Recent seroprevalence research has been used to predict the true prevalence of DENV infection and country-specific dengue endemicity [70,73,74,75,76,77,78,79,80,81,82,83,84,85,86,87,88,89,90,91,92,93,94,95,96,97,98,99,100,101]. The vast majority of these studies found that dengue prevalence and evidence of anti-DENV antibodies from past infection were much higher in number than what was currently reported. One study found that among 80,977 African participants from 2000–2019, seropositivity for DENV was 24.8% in febrile participants and 15% in healthy participants [37]. These sero-epidemiological studies provide additional evidence that dengue in Africa is significantly underreported, and more of a risk to African people and travelers than is currently expected. Figure 2 maps the endemicity and outbreak potential and further identifies countries without reported dengue cases, but with *Aedes* vector spread.

International travel has led to the importation of dengue cases from Africa to dengue-naïve areas or areas with low dengue transmission. These cases, especially when the traveler returned from an area of Africa not considered to be a high risk for dengue transmission, provide more evidence for endemic dengue circulation in African countries. There have been cases of dengue transmission from African countries such as Benin, Côte d’Ivoire, Tanzania, the Democratic Republic of the Congo, Burkina Faso, and Angola to numerous other countries, including mainland China, Italy, Japan, Spain, France, Austria, the Netherlands, Belgium, Norway, Poland, and the United States [102,103,104,105,106,107,108,109,110,111,112,113,114,115,116,117,118,119,120,121,122,123,124,125,126,127]. These populations included people visiting family members or friends, students studying abroad, businesspeople, and military troops [102,103,104,105,106,107,108,109,110,111,112,113,114,115,116,117,118,119,120,121,122,123,124,125,126,127]. Recording cases obtained from international travel to Africa is not just important for estimating endemicity in African countries in the absence of well-established in-country surveillance but is also useful in informing other countries about what precautions are necessary and what risks are to be expected for their citizens traveling to Africa. Additionally, they help to inform healthcare workers in dengue-naïve countries of what diseases their patients may be presenting with in emergency department settings based on their recent travel history.

There is additional evidence that dengue prevalence may be more pronounced in Africa than research currently suggests. The dissemination of the main vectors, *Aedes aegypti* and *Aedes albopictus*, is substantial across the African continent. Research shows these vectors exist widely in sub-Saharan Africa and may be underrecognized due to the vector control strategies that are traditionally focused on *Anopheles* species, which transmit malaria [128]. There is some evidence in laboratory settings that the African strains of *Aedes* are less susceptible to the four DENV subtypes, but more research is needed to determine whether this sufficiently contributes to the explanation of why dengue transmission appears less common in Africa than other locations [30]. A study in Kenya found DENV in 0.4% of mosquito pools, with evidence of transovarial transmission in local mosquitoes [129]. Interviews with a study population of coastal Kenyans revealed a lack of knowledge about the *Aedes* mosquito life cycle and prevention strategies, and a prioritization of *Anopheles* control methods [130]. The paper “*Aedes* Mosquitoes and *Aedes*-Borne Arboviruses in Africa: Current and Future Threats” modeled the potential spread of *Aedes* species in Africa and created maps of the predicted population at risk of infection (PAR) [18]. These maps predict that *Aedes* spread in Africa is far more widespread than is currently predicted or recorded and highlights the importance of adequate recording and data in order to predict future outbreaks. The paper also highlights the fact that recent studies show new *Aedes albopictus* spread in Mali, Morocco, and Mozambique and new *Aedes aegypti* spread in Ghana, Mozambique, and Namibia. As discussed previously, climate change and increased urbanization are also factors that are predicted to increase the *Aedes* populations in Africa. Increases in global warming will create temperature conditions that are more ideal for mosquito larvae, rain will create more opportunities for breeding. Furthermore, urbanization, plastic and tire pollution, and lower socioeconomic development all have the potential to be beneficial for *Aedes* proliferation [8,18,128,131].

There have been questions raised over the true burden of dengue in Africa, especially severe disease [36]. The establishment of endemicity in African countries has been complicated by a lack of adequate reporting. Reasons for this include a lack of adequate surveillance and response systems, including laboratory surveillance methods, insufficient knowledge and awareness of dengue fever disease symptoms and risk, and resource constraints [36,132,133,134]. Another major problem is misdiagnosis and misclassification of febrile disease, especially as malaria or when there is a co-infection of DENV and *Plasmodium* spp. (malaria) [135].

### 3.2. Malaria and Dengue: A Diagnostic Nightmare

Dengue is commonly misdiagnosed due to its non-specific features. In addition, according to the U.S. CDC, around one in four people infected with dengue will show symptoms; the majority of patients are otherwise asymptomatic, which further complicates public health control of dengue [136]. If asymptomatic patients continue to engage in regular day-time behavior, unaware of their infection, female *Aedes* mosquitoes can take a blood meal and transmit the virus, making outbreaks harder to contain and causing transmission to occur at high rates [137]. Seroprevalence studies in various Africa countries of healthy and febrile individuals find high rates of seropositivity, ranging from 0.5% to 73% (determined by measuring IgG antibodies, which is evidence of past infection, and/or IgM, indicating a recent infection), suggesting substantial asymptomatic infection and/or under-reporting or misdiagnosis of clinical cases [70,73,74,75,76,77,78,79,80,81,82,83,84,85,86,87,88,89,90,91,92,93,94,95,96,97,98,99,100,101]. Seroprevalence is used for determining dengue endemicity in African countries otherwise unknown to have local dengue spread. In order to diagnose dengue in Africa, there are a variety of possible tests, including viral genome detection, NS1 antigen tests, IgM and IgG antibody testing via enzyme-linked immunosorbent assays (ELISAs), antibody neutralization tests, rapid antigen or antibody-based diagnostics, and immunofluorescence assays [138]. In Africa, particularly in rural areas, routine testing for arboviruses is uncommon outside of outbreak situations due to issues with transportation, sample and test quality, resource and facility constraints, staff training deficits, and cost [132,135,138], all of which contribute to underdiagnosis and misdiagnosis.

Common dengue symptoms in those who do get sick includes fever with any of the following additional symptoms: nausea, vomiting, rash, and eye, bone, muscle, or joint pain/aches [136]. Severe dengue disease (DHF or DSS, according to the 1997 WHO classification) may manifest as abdominal pain, vomiting (including vomiting blood), hemorrhagic manifestations, and feeling tired or restless, with the most critical feature being plasma leakage that can lead to hypovolemic shock [136]. The WHO 2009 classification specifies dengue without warning signs, dengue with warning signs, and severe dengue (symptoms of which include severe plasma leakage, severe bleeding, or organ failure) [139]. Dengue symptoms are very similar to other endemic diseases in Africa, including viral diseases such as Zika and chikungunya, which are also spread by *Aedes* mosquitoes, Rift Valley fever, yellow fever, and Ebola [140]. However, no disease is as widespread in Africa and seems to cause as much confusion and misdiagnosis as malaria, another mosquito-borne infectious disease.

The WHO estimates that 92% of malaria cases across the world (around 200 million cases) are in the WHO African region [141]. More than 409,000 people died of malaria globally in the year 2019, and most of these deaths were children in sub-Saharan Africa [142]. Presently, 106 countries are considered at risk of malaria transmission, many of which are in the WHO areas of Africa, South East Asia, and the Eastern Mediterranean, as well as the Caribbean and Latin America [143,144].

Malaria, similar to dengue, is a febrile illness, with symptoms that may include fever, chills, general discomfort, headache, nausea/vomiting, diarrhea, abdominal pain, muscle or joint pain, fatigue, rapid breathing or heart rate, and/or cough [145]. These symptoms overlap broadly with dengue. Malaria is well-known among healthcare workers to be a common and expected cause of febrile illness in Africa, and it is largely endemic. Thus, due to widespread malaria, many healthcare workers in Africa assume a presumptive malaria diagnosis when witnessing clinical cases of fever [35,132]. Clinicians may even be aware of dengue as a possible diagnosis, but are uninformed as to the symptoms, risk, and testing [132,133,146]. Research shows that malaria diagnoses are over-diagnosed and can be overestimated by as much as 61% of clinical diagnoses, while more than 70% of febrile illnesses are presumed to be malaria by clinicians when seen in malaria-endemic African countries [35]. A study in Côte d’Ivoire on febrile patients found that among 406 febrile patients who were clinically suspected to have malaria, only 39.4% had a positive thick blood film test, and three patients were positive for dengue following laboratory testing but were not clinically suspected to have dengue [147]. Multiple studies of children in Kenya have found high dengue burden among children with undifferentiated fevers (7.4–41.9%), including evidence of all four DENV serotypes [39,148]. Similar studies in other African countries have ascertained similar results: dengue appears to be more common than estimated, and dengue cases are often misdiagnosed as malaria [149,150,151]. Some research supports the existence of a so-called “malaria-industrial complex”, created in part by malaria control programs and international development organizations in the fight against malaria, that has obscured other febrile illnesses such as dengue and led to misdiagnosis of malaria at high rates due to the focus on malaria as the main cause of fever in sub-Saharan Africa [152]. It is important to note that the problem of misdiagnosis between dengue and malaria is not necessarily restricted to Africa. Any country that has endemic dengue and malaria or has experienced locally acquired outbreaks of both these diseases could be facing the same issue of malaria/dengue misdiagnosis or malaria overestimations.

### 3.3. Malaria and Dengue: The Added Complication of Co-Infections

Another complication of the co-existence of malaria and dengue in Africa is co-infections. Simultaneous infections with the agents of the two diseases have been reported in countries with overlapping prevalence of disease, inside and outside of the African continent. Anopheles mosquitoes that transmit *Plasmodium* bite at night, while *Aedes* mosquitoes bite mostly in the early morning and late afternoon, and these behaviors have implications for co-infection, as a lack of proper disease control methods that address both diseases can leave African populations vulnerable to multiple infections [153].

Multiple co-infections with DENV and *Plasmodium* (mostly *vivax*) have been recorded in India, a country with well-recognized dengue and concurrent malaria activity [154,155,156,157,158,159,160,161,162,163,164]. Case reports and studies among febrile patients revealed that generally, DENV and *Plasmodium* co-infection was associated with more severe symptoms than having either infection alone, including more hemorrhagic manifestations, jaundice, and kidney disfunction [154,155,156,157,158,159,160,161,162,163,164]. In addition to India, reports of *Plasmodium* spp. and DENV co-infection were also reported in China, the Malaysia/Thailand border, Pakistan, Peru, the Brazilian Amazon, Brazil, Haiti, French Guiana, Indonesia, East Timor, Cambodia, Bangladesh, and Japan [159,165,166,167,168,169,170,171,172,173,174,175,176,177,178,179,180,181,182]. In Africa, such co-infections have been reported in Cameroon, Kenya, Nigeria, Ghana, and Senegal [150,183,184,185,186,187,188,189,190,191,192]. In the cited works, co-infection rates in groups of African patients range from 0.6% to as high as 51.5%, and many are co-infections with *Plasmodium* falciparum, given that it is the most common malaria parasite in Africa [39,150,184,185,187,190,191,192]. As previously described, dengue is not well documented in Africa, and cases may be misdiagnosed as a mono-infection of malaria or other febrile illnesses, so there are likely more co-infections across the African continent than what is currently known and described in the literature. These studies in African populations also found mixed evidence that co-infection results in more severe disease in many clinical cases, based on symptoms experienced by the patients enrolled who had either concurrent or mono-infections of DENV/*Plasmodium*, with most patients experiencing more severe disease in cases of concurrent infection compared to mono-infections. The suggested complication of more severe disease due to co-infections is an important condition that clinicians should be aware of in endemic areas where these two diseases may have overlapping epidemiology. Most studies used laboratory parameters such as hemoglobin and platelet levels, *Plasmodium* parasitemia levels, and clinical outcomes (such as duration of fever and/or hospitalization, the need for transfusions, etc.) as methods for analysis.

A systematic review and meta-analysis on the effect of co-infection on malaria severity found that severe malaria was more common among patients co-infected with DENV [193]. A research study in the Brazilian Amazon supported this, finding more severe disease in co-infected patients and lower levels of platelets and hemoglobin [175]. Co-infection exacerbated symptoms of both diseases compared to mono-infection, including fever duration, bleeding, jaundice, renal dysfunction, hepatomegaly, thrombocytopenia, anemia, shock, impaired consciousness, and number of transfusions [156,163,168,193]. There is a very limited amount of information in the literature regarding the biological effects of DENV on *Plasmodium* and vice versa, and more research is needed to elucidate these mechanisms. A study suggested that compared to mono-infections with either agent, co-infection with DENV and *Plasmodium* may be more severe due to the effects of DENV on the endothelium and the resulting increase in vascular permeability, which can cause more severe malaria, but more research is needed to define this relationship [168].

However, another systematic review and meta-analysis on co-infection found that DENV infection may actually decrease the odds of *Plasmodium* infection and parasitemia compared to mono-infection with *Plasmodium*, and that co-infection with DENV was associated with higher platelet and hemoglobin levels in patients [194]. Finally, other studies found no clear differences in severity between mono-infected and co-infected individuals among their sampled patients [165,169]. It is clear that more comprehensive research is needed to reach a conclusion on whether DENV and *Plasmodium* spp. co-infection incurs a greater risk of severe disease than mono-infection, as well as research on possible biological mechanisms for this risk difference, especially given that much of the available research consists of case reports. However, based on what is available currently, there seems to be a relationship that is relevant to clinicians, which provides further evidence for the utility of screening for both diseases among febrile patients.

### 3.4. African Ancestry and Protection from Severe Dengue: Genetics, Social Factors, and Impacts

Risk of severe dengue is associated with multiple potential factors, including the host immune response, host genetic factors, and comorbidities such as diabetes, cardiovascular disease and stroke, respiratory disease, renal disease, and sickle cell disease, as well as co-infections with other infectious diseases [195,196,197]. Delaying care or not seeking care is also associated with more severe outcomes of dengue [195]. Multiple studies also examine genetic ancestry as a risk, which will be discussed in this section. This is relevant to the question of dengue burden in Africa, as the literature suggests that some level of protection may exist against severe dengue among those with African ancestry. Whether this is a true protective genetic relationship or a result of other factors such as misclassification, socioeconomic conditions, health-seeking behavior, and/or medical racism is a subject of controversy among researchers. If there is truly a protective effect, then severe dengue may not be a high priority concern among African populations. If this is a falsely identified protective effect confounded by other social factors, then clinicians may neglect to consider dengue as a possible diagnosis for sick patients in these populations and may misdiagnose them. Proper care could be delayed, or mosquito abatement techniques abandoned, which would negatively affect entire communities [198]. Therefore, it is of the utmost importance to ascertain this question of protection.

It is important to distinguish between genetic ancestry and race for the context of this paper. Race is a social construct, and biological characteristics and genetics do not differ fundamentally among different socially ascribed races or skin colors [199,200]. This paper makes no attempt to suggest that there is a fundamental biological component to race, and the authors understand that the idea of race is constructed, fluid, and not based in biological science. Such biological arguments for race have historically been used to justify genocide, residential segregation, assertions of biological inferiority, and other racist practices [201]. Oftentimes, perceived racial differences in health outcomes are due to confounding variables such as socioeconomic differences, institutional racism, or bias on behalf of healthcare providers. The emphasis, therefore, should be on why certain racial groups are disproportionately affected by inequities and how this relates to healthcare outcomes, and not on how people of different skin color may be biologically different.

However, research has identified situations where genetic ancestry, based on changes in DNA at the epigenetic level or due to varying evolutionary pressures, may relate to differing healthcare outcomes. For example, sickle cell disease tends to have a higher incidence in populations whose ancestry traces back to areas with high malaria incidence, as the sickle cell trait is protective against malaria [202]. As malaria is more widespread in sub-Saharan African populations than any other group, sickle cell disease tends to become associated with Black patients. Both self-identified race and ethnicity and genetic ancestry are important to consider when treating patients or developing interventions, as bias, discrimination, poverty, and healthcare access may be just as important to consider when assessing a patient’s risk as any genetic ancestral risk component would be [201]. The following section will examine the literature describing potential genetic components affecting risk for DHF/DSS, with the major caveat that some of these studies have focused on Black populations/patients without specifically examining ancestry itself. In these situations, racial classifications may differ based on what a specific provider ascribes a patient’s race to be in a study compared to how the patients self-identify, and these classifications may differ by country, culture, or context, were the study to be repeated. Self-ascribed racial categories may also fail to examine the role of socio-cultural factors that impact dengue severity.

Various studies excluding racial/ethnic/ancestral categorization have suggested the existence of genetic components that modify an individual’s risk of progressing to severe dengue disease. The review “Host Genetics and Dengue Fever” found that genes affecting DENV uptake (e.g., FcγRIIA, CD209, and CLEC5A) may affect the immune response intensity in a patient and therefore dengue severity, as severity is often a result of host immune response overactivation [203]. Research has also shown that some serotype-specific variants at oligoadenylate synthetase (OAS) family genes affect dengue severity, and that polymorphisms in a series of genes, including MICB, PLCE1, TNF, TPSAB1, and IL10, have been associated with dengue outcomes and severity level in multiple studies [203]. Different human leukocyte antigen (HLA) groups have also been shown to confer either protection against or risk of developing severe dengue manifestations, including HLA-A 0203, 0207, A11, B-15, B-44, B-46, B-48, B-51, and B-52, which have different effects in various ethnic groups [204,205]. Some HLA types associated with more severe dengue disease include but are not limited to HLA-A*31, A*01, A*26, A*31, A*68, A*32, and A*30, as well as B*44, B*15, and B*35:01 [206]. Several HLA types associated with protection from severe disease include HLA-DR*9, DR*12, B*07, and DR*13 [207]. Additionally, AB blood group has been shown to be a risk factor for DHF/DSS [208]. Other studies outline specific genetic risks for dengue severity based on genome-wide association studies, including analyses for multiple ancestries [209].

Multiple studies suggest a relationship between African ancestry and protection from severe dengue disease. In Cuban populations, where the population is typically a mixture of African, European, and Native American ancestry, African descendancy has been associated with protection [210]. A study of risk factors for DHF/DSS in admixed Cuban populations found that candidate genes OSBPL10 and RXRA are expressed differently during dengue disease progression [210]. A decrease in OSBPL10 expression, which was significantly lower in African descendants, was associated with a decrease in DENV-2 replication and therefore decreased disease severity. Different SNPs regulate RXRA transcription between African and European populations, which regulates important immune functions particularly in macrophages [210]. Following a 1981 dengue epidemic in Cuba, clinicians noticed that Black individuals had a significantly lower frequency of DHF/DSS compared to White patients but did not have an explanation of why this phenomenon occurred and did not consider socioeconomic status or health behavior [211]. Another study on this 1981 epidemic and a 1977 dengue epidemic in Cuba found that White individuals had stronger and more cross-reactive DENV-specific memory CD4^+^ T lymphocyte proliferation and interferon (IFN)-γ release compared to Black individuals, which could contribute to the immunopathogenesis that leads to severe dengue and may help to explain why Black individuals had less severe dengue disease [212]. Researchers investigating ADE of viral replication of DENV-2 in peripheral blood mononuclear cells (PBMCs) of ‘Cubans of White and Black descent’ found that DENV-2 did not replicate well in PBMCs of Black individuals ex vivo, regardless of the presence of anti-DENV antibodies, but increased viral proliferation was found in the PBMCs of White individuals, which could partially explain the increased risk of DHF/DSS [213]. Studies in Cuban populations suggest a genetic protective effect in African descent populations, but the descriptive clinical studies may have conflated race and ancestry and failed to consider social explanations for the decreased cases of dengue severity in Black patients.

A genetics meta-analysis on seven genes associated with dengue severity risk found that sub-Saharan African populations and descendants of these populations are most protected against DHF/DSS compared to other ancestral groups and that Southeast/Northeast Asians are the least protected [214]. Similarly, the “Host genetics and dengue fever” review discussed above also found that African ancestry is associated with protection from severe dengue [203]. In Haiti, researchers studying DHF in Haitian children found that while 85% of the children studied had antibodies to at least two DENV serotypes, there were no DHF reported by pediatricians, but DHF cases did occur in United States/United Nations military personnel in Haiti, which they attributed as evidence for dengue resistance genes in Black populations [215]. In a study on Columbian populations with dengue, for every 1% increase in African ancestry, there was a more significant protective effect against severe dengue, dengue hemorrhagic fever, and hemorrhage occurrence, and a decrease “from 100% to 0% African ancestry” increased the odds ratio for severe dengue by 44-fold, DHF by 24-fold, and hemorrhagic occurrence by 20-fold [216]. In Dar es Salaam, Tanzania, a study of native and expatriate populations in an outpatient clinic found that amongst patients with the same DENV serotype, African ancestry was protective against severe dengue, due to environmental or genetic host factors, and suggested that this mild course of disease may provide explanation for the under-reporting of dengue in Africa and the frequent misdiagnosis of dengue as malaria [217]. Additional studies have found significant findings with similar evidence of protective effects in populations of African descent [218,219,220,221].

Current evidence points to a genetic protective effect against severe dengue manifestations in populations of African descent. Ultimately, more research is needed, including a comprehensive meta-analysis, to determine whether this evidence holds true amongst larger populations, as many of these research studies were specific to patients presenting to hospitals or clinics in different geographic locations where people of African descent reside. Additionally, there have not been many studies focusing on patients specifically residing in Africa and their susceptibility to dengue, outside of the Tanzania study. A study on genetic ancestry and income also found that DHF diagnosis was associated with higher income level (and was independently negatively associated with African ancestry) [218]. This highlights the potential effects of socioeconomic factors on this perceived relationship, as DHF is typically diagnosed in hospitals, which may only be accessible to people of higher income level and with better access to care [218]. If only patients presenting to hospitals are being diagnosed, then this may mean that DHF is not as uncommon among African ancestral groups as is currently expected, although ex vivo cell studies seemed to still indicate a relationship. Additionally, a review on causes of dengue mortality found that low education levels, poverty, delay of care or absence of care-seeking, rural residence, barriers to health access, low government expenditures on health, low health staff knowledge of dengue, and poor dengue surveillance systems were all related to higher dengue mortality [195]. Ultimately, clinicians should be careful to not dismiss severe dengue as a diagnosis among African descent populations, as cases still do occur and could be misdiagnosed or mistreated if an association with decreased severity is assumed in all cases. Additionally, this relationship does not suggest that DENV is not circulating among those of African descent, but rather that severe cases are less common (although they do exist, and outbreaks of DHF in African patients do still occur, even if rare) [222]. Therefore, public health control measures in populations of people with high degrees of African descent are still necessary to ensure overall health and well-being. Dengue transmission, infection, and clinical severity are multifactorial, and there is not one overriding factor to explain differences in clinical presentation.

## 4. Conclusions and Next Steps

This review highlights important considerations for dengue disease in Africa, including a changing epidemiology and underestimation of disease burden, issues with malaria misdiagnosis and co-infections, and limited evidence of genetic protection from severe dengue disease in populations of African descent.

A review of the literature indicated that DENV prevalence in African countries and populations may be more widespread than reported data suggest. Recent outbreaks indicate an increasing problem in East Africa, and DENV may be circulating undiagnosed in West Africa as well [131,223]. Additionally, *Aedes* mosquitoes, the vectors for DENV, appear to be increasing in geographic dissemination and number. Climate change, urbanization, plastic pollution, and population growth are all factors expected to make the dengue situation in Africa worse in the near future [8,128,131]. Dengue in Africa therefore deserves more attention in terms of public health interventions such as vector control, dengue disease surveillance and rapid, inexpensive diagnostic testing, as well as training of clinicians in dengue symptoms/diagnosis and supportive treatment options.

Dengue misdiagnosis is a problem in Africa, especially due to the typical non-specific clinical presentation of dengue. Additionally, current literature suggests that the widespread endemicity of malaria in Africa complicates the diagnosis of dengue, as most febrile illnesses are assumed to be malaria. This is a particular problem for vector control strategies. The typical vector control strategies for the malaria-spreading *Anopheles* mosquitoes, such as insecticide-treated bednets and indoor insecticide spraying, would not be as effective against *Aedes* mosquitoes, which typically bite during the day [128].

Research into a protective genetic component against severe dengue in African descent populations is suggestive of the existence of such a relationship, but a meta-analysis and further studies should be conducted to strengthen this association in various populations. Additionally, more studies should address socioeconomic factors that may contribute to this relationship and better delineate between self-ascribed or researcher-ascribed racial categories and actual genetic ancestry information.

The main finding of this review is that based on the evidence of dengue and *Aedes* dissemination, recent outbreaks, widespread seropositivity, climate change factors, malaria misdiagnosis, *Plasmodium* and DENV co-infection, and questions around genetic protection from severe dengue versus social determinants of health barriers and misdiagnosis, Africa should not be ignored when it comes to dengue. More research should be performed to examine the relationship of African ancestry and dengue severity risk. In the meantime, clinicians should be aware to considering DHF as a possible diagnosis, as some patients of African descent can develop this disease despite potential general genetic protection. Proper surveillance systems, laboratory testing facilities, clinician diagnostic training materials for non-malarial febrile illnesses, and vector control strategies also urgently need to be put in place in African countries, including those without currently reported dengue cases but with current or predicted *Aedes* spread, in order to reduce the burden of this disease. 

## Figures and Tables

**Table 1:** Outbreaks of DENV in Africa in the past decade (2011–2021).

Subregion of Africa	Country	Outbreaks Recorded in the Last 10 Years (by Year)	U.S. CDC Level of Risk
**North Africa**	**Sudan**	**2013 [42,43], 2014–2015 [44], 2017 [42]**	Frequent/Continuous
	**Egypt**	**2015 [45]**	Sporadic/Uncertain
	Algeria	None reported in the past decade	Not listed
	Libya	None reported in the past decade	Not listed
	Morocco	None reported in the past decade	Not listed
	Tunisia	None reported in the past decade	Not listed
**East Africa**	**Eritrea**	**2014, 2015 [46]**	Frequent/Continuous
	**Djibouti**	**2011–2014 [47]**	Frequent/Continuous
	**Ethiopia**	**2013, 2014, 2015, 2016 [48,49,50]**	Frequent/Continuous
	**Kenya**	**2011, 2013–2014, 2017–2018 [51,52,53,54]**	Frequent/Continuous
	**Somalia**	**2011 [54,55]**	Frequent/Continuous
	**Tanzania**	**2014, 2018, 2019 [56,57]**	Frequent/Continuous
	**Mauritius**	**2019 [58]**	Sporadic/Uncertain
	**Mozambique**	**2014 [59], 2015 [43]**	Sporadic/Uncertain
	**Seychelles**	**2015–2017 [60,61]**	Sporadic/Uncertain
	Burundi	None reported in the past decade	Sporadic/Uncertain
	Comoros	None reported in the past decade	Sporadic/Uncertain
	Madagascar	None reported in the past decade	Sporadic/Uncertain
	Malawi	None reported in the past decade	Sporadic/Uncertain
	Rwanda	None reported in the past decade	Sporadic/Uncertain
	South Sudan	None reported in the past decade	Sporadic/Uncertain
	Uganda	None reported in the past decade	Sporadic/Uncertain
	Zambia	None reported in the past decade	Sporadic/Uncertain
	Zimbabwe	None reported in the past decade	Sporadic/Uncertain
**Central Africa**	**Angola**	**2013 [62,63]**	Sporadic/Uncertain
	**Cameroon**	**2017 [64]**	Sporadic/Uncertain
	Central African Republic	None reported in the past decade	Sporadic/Uncertain
	Chad	None reported in the past decade	Sporadic/Uncertain
	Democratic Republic of the Congo	None reported in the past decade	Sporadic/Uncertain
	Republic of the Congo	None reported in the past decade	Sporadic/Uncertain
	Equatorial Guinea	None reported in the past decade	Sporadic/Uncertain
	Gabon	None reported in the past decade	Sporadic/Uncertain
	São Tomé and Príncipe	None reported in the past decade	Sporadic/Uncertain
**West Africa**	**Burkina Faso**	**2013, 2016–2017 [65,66]**	Frequent/Continuous
	**Ivory Coast** (**Côte d’Ivoire**)	**2017, 2019 [58,67]**	Sporadic/Uncertain
	**Senegal**	**2015, 2018–2019 [68,69]**	Sporadic/Uncertain
	Benin	None reported in the past decade	Sporadic/Uncertain
	Cape Verde	None reported in the past decade	Sporadic/Uncertain
	Gambia	None reported in the past decade	Sporadic/Uncertain
	Ghana	None reported in the past decade	Sporadic/Uncertain
	Guinea	None reported in the past decade	Sporadic/Uncertain
	Guinea-Bissau	None reported in the past decade	Sporadic/Uncertain
	Liberia	None reported in the past decade	Sporadic/Uncertain
	Mali	None reported in the past decade	Sporadic/Uncertain
	Niger	None reported in the past decade	Sporadic/Uncertain
	Nigeria	None reported in the past decade	Sporadic/Uncertain
	Sierra Leone	None reported in the past decade	Sporadic/Uncertain
	Togo	None reported in the past decade	Sporadic/Uncertain
	Mauritania	None reported in the past decade	Not listed
**Southern Africa**	Namibia	None reported in the past decade	Sporadic/Uncertain
	Botswana	None reported in the past decade	Not listed
	Eswatini (Swaziland)	None reported in the past decade	Not listed
	Lesotho	None reported in the past decade	Not listed
	South Africa	None reported in the past decade	Not listed

Note: Bolded countries denote outbreaks in the past decade.

## References

[1] Neglected Tropical Diseases. https://www.who.int/news-room/q-a-detail/neglected-tropical-diseases.

[2] About Dengue: What You Need to Know. https://www.cdc.gov/dengue/about/index.html.

[3] Cattarino L., Rodriguez-Barraquer I., Imai N., Cummings D.A.T., Ferguson N.M. (2020). Mapping global variation in dengue transmission intensity. Sci. Transl. Med..

[4] Gubler D.J., Clark G.G. (1995). Dengue/dengue hemorrhagic fever: The emergence of a global health problem. Emerg. Infect. Dis..

[5] Bhatt S., Gething P.W., Brady O.J., Messina J.P., Farlow A.W., Moyes C.L., Drake J.M., Brownstein J.S., Hoen A.G., Sankoh O. (2013). The global distribution and burden of dengue. Nature.

[6] Gwee S.X.W., St John A.L., Gray G.C., Pang J. (2021). Animals as potential reservoirs for dengue transmission: A systematic review. One Health.

[7] Back A.T., Lundkvist A. (2013). Dengue viruses—An overview. Infect. Ecol. Epidemiol..

[8] Murray N.E., Quam M.B., Wilder-Smith A. (2013). Epidemiology of dengue: Past, present and future prospects. Clin. Epidemiol..

[9] Guzman M.G., Harris E. (2015). Dengue. Lancet.

[10] Dengue and Severe Dengue. https://www.who.int/news-room/fact-sheets/detail/dengue-and-severe-dengue.

[11] World Health Organization (1997). Dengue Haemorrhagic Fever: Diagnosis, Treatment, Prevention and Control.

[12] Kalayanarooj S. (2011). Clinical Manifestations and Management of Dengue/DHF/DSS. Trop. Med. Health.

[13] World Health Organization (2009). Dengue Guidelines for Diagnosis, Treatment, Prevention and Control: New Edition.

[14] Gubler D.J. (2002). Epidemic dengue/dengue hemorrhagic fever as a public health, social and economic problem in the 21st century. Trends Microbiol..

[15] Chotpitayasunondh T. (2012). Introduction on the global dengue epidemiological burden. Int. J. Infect. Dis..

[16] Braack L., Gouveia De Almeida A.P., Cornel A.J., Swanepoel R., De Jager C. (2018). Mosquito-borne arboviruses of African origin: Review of key viruses and vectors. Parasites Vectors.

[17] Pierson T.C., Diamond M.S. (2020). The continued threat of emerging flaviviruses. Nat. Microbiol..

[18] Weetman D., Kamgang B., Badolo A., Moyes C.L., Shearer F.M., Coulibaly M., Pinto J., Lambrechts L., McCall P.J. (2018). Aedes Mosquitoes and Aedes-Borne Arboviruses in Africa: Current and Future Threats. Int. J. Environ. Res. Public Health.

[19] Guzman M.G., Alvarez M., Halstead S.B. (2013). Secondary infection as a risk factor for dengue hemorrhagic fever/dengue shock syndrome: An historical perspective and role of antibody-dependent enhancement of infection. Arch. Virol..

[20] Katzelnick L.C., Gresh L., Halloran M.E., Mercado J.C., Kuan G., Gordon A., Balmaseda A., Harris E. (2017). Antibody-dependent enhancement of severe dengue disease in humans. Science.

[21] Katzelnick L.C., Narvaez C., Arguello S., Lopez Mercado B., Collado D., Ampie O., Elizondo D., Miranda T., Bustos Carillo F., Mercado J.C. (2020). Zika virus infection enhances future risk of severe dengue disease. Science.

[22] Vaccines and Immunization: Dengue. https://www.who.int/news-room/q-a-detail/dengue-vaccines.

[23] Wen J., Shresta S. (2019). Antigenic cross-reactivity between Zika and dengue viruses: Is it time to develop a universal vaccine?. Curr. Opin. Immunol..

[24] Caldwell J.M., Labeaud A.D., Lambin E.F., Stewart-Ibarra A.M., Ndenga B.A., Mutuku F.M., Krystosik A.R., Ayala E.B., Anyamba A., Borbor-Cordova M.J. (2021). Climate predicts geographic and temporal variation in mosquito-borne disease dynamics on two continents. Nat. Commun..

[25] Tjaden N.B., Thomas S.M., Fischer D., Beierkuhnlein C. (2013). Extrinsic Incubation Period of Dengue: Knowledge, Backlog, and Applications of Temperature Dependence. PLoS Negl. Trop. Dis..

[26] Githeko A.K., Lindsay S.W., Confalonieri U.E., Patz J.A. (2000). Climate change and vector-borne diseases: A regional analysis. Bull. World Health Org..

[27] Nosrat C., Altamirano J., Anyamba A., Caldwell J.M., Damoah R., Mutuku F., Ndenga B., Labeaud A.D. (2021). Impact of recent climate extremes on mosquito-borne disease transmission in Kenya. PLoS Negl. Trop. Dis..

[28] Ebi K.L., Nealon J. (2016). Dengue in a changing climate. Environ. Res.

[29] Krystosik A., Njoroge G., Odhiambo L., Forsyth J.E., Mutuku F., LaBeaud A.D. (2019). Solid Wastes Provide Breeding Sites, Burrows, and Food for Biological Disease Vectors, and Urban Zoonotic Reservoirs: A Call to Action for Solutions-Based Research. Front. Public Health.

[30] Were F. (2012). The dengue situation in Africa. Paediatr. Int. Child Health.

[31] Harapan H., Michie A., Sasmono R.T., Imrie A. (2020). Dengue: A Minireview. Viruses.

[32] Mulligan K., Dixon J., Joanna Sinn C.-L., Elliott S.J. (2015). Is dengue a disease of poverty? A systematic review. Pathog. Glob. Health.

[33] Patel N. (2018). Figure of the Week: Understanding Poverty in Africa.

[34] Shepard D.S., Undurraga E.A., Halasa Y.A., Stanaway J.D. (2016). The global economic burden of dengue: A systematic analysis. Lancet Infect. Dis..

[35] Amarasinghe A., Kuritsk J.N., Letson G.W., Margolis H.S. (2011). Dengue virus infection in Africa. Emerg. Infect. Dis..

[36] Jaenisch T., Junghanss T., Wills B., Brady O.J., Eckerle I., Farlow A., Hay S.I., McCall P.J., Messina J.P., Ofula V. (2014). Dengue expansion in Africa-not recognized or not happening?. Emerg. Infect. Dis..

[37] Simo F.B.N., Bigna J.J., Kenmoe S., Ndangang M.S., Temfack E., Moundipa P.F., Demanou M. (2019). Dengue virus infection in people residing in Africa: A systematic review and meta-analysis of prevalence studies. Sci. Rep..

[38] Humphrey J.M., Cleton N.B., Reusken C.B., Glesby M.J., Koopmans M.P., Abu-Raddad L.J. (2016). Dengue in the Middle East and North Africa: A Systematic Review. PLoS Negl. Trop. Dis..

[39] Shah M., Ndenga B., Mutuku F., Vu D., Grossi-Soyster E., Okuta V., Ronga C., Chebii P., Maina P., Jembe Z. (2020). High Dengue Burden and Circulation of 4 Virus Serotypes among Children with Undifferentiated Fever, Kenya, 2014–2017. Emerg. Infect. Dis. J..

[40] Standard Country or Area Codes for Statistical Use. https://unstats.un.org/unsd/methodology/m49/.

[41] Dengue around the World. https://www.cdc.gov/dengue/areaswithrisk/around-the-world.html.

[42] Dengue Fever—Republic of the Sudan, 22 November 2019. https://reliefweb.int/report/sudan/dengue-fever-republic-sudan-22-november-2019.

[43] Baba M., Villinger J., Masiga D.K. (2016). Repetitive dengue outbreaks in East Africa: A proposed phased mitigation approach may reduce its impact. Rev. Med. Virol..

[44] Ahmed A., Ali Y., Elmagboul B., Mohamed O., Elduma A., Bashab H., Mahamoud A., Khogali H., Elaagip A., Higazi T. (2019). Dengue Fever in the Darfur Area, Western Sudan. Emerg. Infect. Dis..

[45] Dengue Fever—Egypt. https://www.who.int/emergencies/disease-outbreak-news/item/12-november-2015-dengue-en.

[46] Usman A., Ball J.D., Rojas D.P., Berhane A., Ghebrat Y., Mebrahtu G., Gebresellasie A., Zehaie A., Mufunda J., Liseth O. (2016). Dengue fever outbreaks in Eritrea, 2005–2015: A case for strengthening surveillance, control and reporting. Glob. Health Res. Policy.

[47] Le Gonidec E., Maquart M., Duron S., Savini H., Cazajous G., Vidal P.O., Chenilleau M.C., Roseau J.B., Benois A., Dehan C. (2016). Clinical Survey of Dengue Virus Circulation in the Republic of Djibouti between 2011 and 2014 Identifies Serotype 3 Epidemic and Recommends Clinical Diagnosis Guidelines for Resource Limited Settings. PLoS Negl. Trop. Dis..

[48] Degife L.H., Worku Y., Belay D., Bekele A., Hailemariam Z. (2019). Factors associated with dengue fever outbreak in Dire Dawa administration city, October, 2015, Ethiopia—Case control study. BMC Public Health.

[49] Gutu M.A., Bekele A., Seid Y., Mohammed Y., Gemechu F., Woyessa A.B., Tayachew A., Dugasa Y., Gizachew L., Idosa M. (2021). Another dengue fever outbreak in Eastern Ethiopia-An emerging public health threat. PLoS Negl. Trop. Dis..

[50] Salah Y.M.A.a.A.A. (2016). Epidemiology of Dengue Fever in Ethiopian Somali Region: Retrospective Health Facility Based Study. Cent. Afr. J. Public Health.

[51] Ellis E.M., Neatherlin J.C., Delorey M., Ochieng M., Mohamed A.H., Mogeni D.O., Hunsperger E., Patta S., Gikunju S., Waiboic L. (2015). A household serosurvey to estimate the magnitude of a dengue outbreak in Mombasa, Kenya, 2013. PLoS Negl. Trop. Dis..

[52] Langat S.K., Eyase F.L., Berry I.M., Nyunja A., Bulimo W., Owaka S., Ofula V., Limbaso S., Lutomiah J., Jarman R. (2020). Origin and evolution of dengue virus type 2 causing outbreaks in Kenya: Evidence of circulation of two cosmopolitan genotype lineages. Virus Evol..

[53] Lutomiah J., Barrera R., Makio A., Mutisya J., Koka H., Owaka S., Koskei E., Nyunja A., Eyase F., Coldren R. (2016). Dengue Outbreak in Mombasa City, Kenya, 2013–2014: Entomologic Investigations. PLoS Negl. Trop. Dis..

[54] Obonyo M., Fidhow A., Ofula V. (2018). Investigation of laboratory confirmed Dengue outbreak in North-eastern Kenya, 2011. PLoS ONE.

[55] Bosa H.K., Montgomery J.M., Kimuli I., Lutwama J.J. (2014). Dengue fever outbreak in Mogadishu, Somalia 2011: Co-circulation of three dengue virus serotypes. Int. J. Infect. Dis..

[56] Chipwaza B., Sumaye R.D., Weisser M., Gingo W., Yeo N.K., Amrun S.N., Okumu F.O., Ng L.F.P. (2021). Occurrence of 4 Dengue Virus Serotypes and Chikungunya Virus in Kilombero Valley, Tanzania, During the Dengue Outbreak in 2018. Open Forum Infect. Dis..

[57] Mboera L.E., Mweya C.N., Rumisha S.F., Tungu P.K., Stanley G., Makange M.R., Misinzo G., De Nardo P., Vairo F., Oriyo N.M. (2016). The Risk of Dengue Virus Transmission in Dar es Salaam, Tanzania during an Epidemic Period of 2014. PLoS Negl. Trop. Dis..

[58] World Health Organization Regional Office for Africa-Weekly Bulletin on Outbreaks and Other Emergencies: Week 27: 01–07 July 2019. https://apps.who.int/iris/bitstream/handle/10665/325777/OEW27-0107072019.pdf.

[59] Massangaie M., Pinto G., Padama F., Chambe G., da Silva M., Mate I., Chirindza C., Ali S., Agostinho S., Chilaule D. (2016). Clinical and Epidemiological Characterization of the First Recognized Outbreak of Dengue Virus-Type 2 in Mozambique, 2014. Am. J. Trop. Med. Hyg..

[60] Lustig Y., Wolf D., Halutz O., Schwartz E. (2017). An outbreak of dengue virus (DENV) type 2 Cosmopolitan genotype in Israeli travellers returning from the Seychelles, April 2017. Eurosurveillance.

[61] Seychelles: Dengue Outbreak Emergency Plan of Action Final Report. https://reliefweb.int/report/seychelles/seychelles-dengue-outbreak-emergency-plan-action-final-report-dref-operation-n.

[62] Mazaba-Liwewe M.L., Siziya S., Monze M., Mweene-Ndumba I., Masaninga F., Songolo P., Malama C., Chizema E., Mwaba P., Babaniyi O.A. (2014). First sero-prevalence of dengue fever specific immunoglobulin G antibodies in Western and North-Western provinces of Zambia: A population based cross sectional study. Virol. J..

[63] Sharp T.M., Moreira R., Soares M.J., Miguel da Costa L., Mann J., DeLorey M., Hunsperger E., Munoz-Jordan J.L., Colon C., Margolis H.S. (2015). Underrecognition of Dengue during 2013 Epidemic in Luanda, Angola. Emerg. Infect. Dis..

[64] Nemg Simo F.B., Sado Yousseu F.B., Evouna Mbarga A., Bigna J.J., Melong A., Ntoude A., Kamgang B., Bouyne R., Moundipa Fewou P., Demanou M. (2018). Investigation of an Outbreak of Dengue Virus Serotype 1 in a Rural Area of Kribi, South Cameroon: A Cross-Sectional Study. Intervirology.

[65] Im J., Balasubramanian R., Ouedraogo M., Wandji Nana L.R., Mogeni O.D., Jeon H.J., van Pomeren T., Haselbeck A., Lim J.K., Prifti K. (2020). The epidemiology of dengue outbreaks in 2016 and 2017 in Ouagadougou, Burkina Faso. Heliyon.

[66] Tarnagda Z., Cissé A., Bicaba B.W., Diagbouga S., Sagna T., Ilboudo A.K., Tialla D., Lingani M., Sondo K.A., Yougbaré I. (2018). Dengue Fever in Burkina Faso, 2016. Emerg. Infect. Dis. J..

[67] Dengue Fever—Côte d’Ivoire. https://www.who.int/emergencies/disease-outbreak-news/item/04-august-2017-dengue-cote-d-ivoire-en.

[68] Dieng I., Cunha M., Diagne M.M., Sembene P.M., Zanotto P.M.A., Faye O., Faye O., Sall A.A. (2021). Origin and Spread of the Dengue Virus Type 1, Genotype V in Senegal, 2015–2019. Viruses.

[69] Gaye A., Ndiaye T., Sy M., Deme A.B., Thiaw A.B., Sene A., Ndiaye C., Diedhiou Y., Mbaye A.M., Ndiaye I. (2021). Genomic investigation of a dengue virus outbreak in Thiès, Senegal, in 2018. Sci. Rep..

[70] Elduma A.H., LaBeaud A.D., Plante J.A., Plante K.S., Ahmed A. (2020). High Seroprevalence of Dengue Virus Infection in Sudan: Systematic Review and Meta-Analysis. Trop. Med. Infect. Dis..

[71] Lim J.K., Matendechero S.H., Alexander N., Lee J.S., Lee K.S., Namkung S., Andia E., Oyembo N., Lim S.K., Kanyi H. (2020). Clinical and epidemiologic characteristics associated with dengue fever in Mombasa, Kenya. Int. J. Infect. Dis..

[72] Mwanyika G.O., Mboera L.E.G., Rugarabamu S., Ngingo B., Sindato C., Lutwama J.J., Paweska J.T., Misinzo G. (2021). Dengue Virus Infection and Associated Risk Factors in Africa: A Systematic Review and Meta-Analysis. Viruses.

[73] Adam A., Schüttoff T., Reiche S., Jassoy C. (2018). High seroprevalence of dengue virus indicates that dengue virus infections are frequent in central and eastern Sudan. Trop. Med. Int. Health.

[74] Botros B.A., Watts D.M., Soliman A.K., Salib A.W., Moussa M.I., Mursal H., Douglas C., Farah M. (1989). Serological evidence of dengue fever among refugees, Hargeysa, Somalia. J. Med. Virol..

[75] Budodo R.M., Horumpende P.G., Mkumbaye S.I., Mmbaga B.T., Mwakapuja R.S., Chilongola J.O. (2020). Serological evidence of exposure to Rift Valley, Dengue and Chikungunya Viruses among agropastoral communities in Manyara and Morogoro regions in Tanzania: A community survey. PLoS Negl. Trop. Dis..

[76] Chepkorir E., Tchouassi D.P., Konongoi S.L., Lutomiah J., Tigoi C., Irura Z., Eyase F., Venter M., Sang R. (2019). Serological evidence of Flavivirus circulation in human populations in Northern Kenya: An assessment of disease risk 2016–2017. Virol. J..

[77] Chisenga C.C., Bosomprah S., Musukuma K., Mubanga C., Chilyabanyama O.N., Velu R.M., Kim Y.C., Reyes-Sandoval A., Chilengi R. (2020). Sero-prevalence of arthropod-borne viral infections among Lukanga swamp residents in Zambia. PLoS ONE.

[78] Collenberg E., Ouedraogo T., Ganamé J., Fickenscher H., Kynast-Wolf G., Becher H., Kouyaté B., Kräusslich H.-G., Sangaré L., Tebit D.M. (2006). Seroprevalence of six different viruses among pregnant women and blood donors in rural and urban Burkina Faso: A comparative analysis. J. Med. Virol..

[79] Elaagip A., Alsedig K., Altahir O., Ageep T., Ahmed A., Siam H.A., Samy A.M., Mohamed W., Khalid F., Gumaa S. (2020). Seroprevalence and associated risk factors of Dengue fever in Kassala state, eastern Sudan. PLoS Negl. Trop. Dis..

[80] Eshetu D., Shimelis T., Nigussie E., Shumie G., Chali W., Yeshitela B., Assefa A., Gadisa E. (2020). Seropositivity to dengue and associated risk factors among non-malarias acute febrile patients in Arba Minch districts, southern Ethiopia. BMC Infect. Dis..

[81] Grossi-Soyster E.N., Cook E.A.J., de Glanville W.A., Thomas L.F., Krystosik A.R., Lee J., Wamae C.N., Kariuki S., Fevre E.M., LaBeaud A.D. (2017). Serological and spatial analysis of alphavirus and flavivirus prevalence and risk factors in a rural community in western Kenya. PLoS Negl. Trop. Dis..

[82] Guyer B. (1972). Serological survey for arboviruses in Igbo-Ora, western Nigeria. Ann. Trop. Med. Parasitol..

[83] Himatt S., Osman K.E., Okoued S.I., Seidahmed O.E., Beatty M.E., Soghaier M.A., Elmusharaf K. (2015). Sero-prevalence of dengue infections in the Kassala state in the eastern part of the Sudan in 2011. J. Infect. Public Health.

[84] Hortion J., Mutuku F.M., Eyherabide A.L., Vu D.M., Boothroyd D.B., Grossi-Soyster E.N., King C.H., Ndenga B.A., LaBeaud A.D. (2019). Acute Flavivirus and Alphavirus Infections among Children in Two Different Areas of Kenya, 2015. Am. J. Trop. Med. Hyg..

[85] Hussen M.O., Sayed A.S.M., Abushahba M.F.N. (2020). Sero-epidemiological study on Dengue fever virus in humans and camels at Upper Egypt. Vet. World.

[86] Inziani M., Adungo F., Awando J., Kihoro R., Inoue S., Morita K., Obimbo E., Onyango F., Mwau M. (2020). Seroprevalence of yellow fever, dengue, West Nile and chikungunya viruses in children in Teso South Sub-County, Western Kenya. Int. J. Infect. Dis..

[87] Kuniholm M.H., Wolfe N.D., Huang C.Y., Mpoudi-Ngole E., Tamoufe U., LeBreton M., Burke D.S., Gubler D.J. (2006). Seroprevalence and distribution of Flaviviridae, Togaviridae, and Bunyaviridae arboviral infections in rural Cameroonian adults. Am. J. Trop. Med. Hyg..

[88] Lim J.K., Carabali M., Lee J.S., Lee K.S., Namkung S., Lim S.K., Ridde V., Fernandes J., Lell B., Matendechero S.H. (2018). Evaluating dengue burden in Africa in passive fever surveillance and seroprevalence studies: Protocol of field studies of the Dengue Vaccine Initiative. BMJ Open.

[89] Mease L.E., Coldren R.L., Musila L.A., Prosser T., Ogolla F., Ofula V.O., Schoepp R.J., Rossi C.A., Adungo N. (2011). Seroprevalence and distribution of arboviral infections among rural Kenyan adults: A cross-sectional study. Virol. J..

[90] Mengesha Tsegaye M., Beyene B., Ayele W., Abebe A., Tareke I., Sall A., Yactayo S., Shibeshi M.E., Staples E., Belay D. (2018). Sero-prevalence of yellow fever and related Flavi viruses in Ethiopia: A public health perspective. BMC Public Health.

[91] Morrill J.C., Johnson B.K., Hyams C., Okoth F., Tukei P.M., Mugambi M., Woody J. (1991). Serological evidence of arboviral infections among humans of coastal Kenya. J. Trop. Med. Hyg..

[92] Muianga A., Falk K., Oludele J., Pinto G., Ali S., Tivane A.T., Galano G., Gudo E.S., Lagerqvist N. (2016). Serological and molecular investigation of dengue, chikungunya and rift valey fever in febrile and non-febrile patients from northern Mozambique during Dengue outbreak, 2014. Int. J. Infect. Dis..

[93] Ochieng C., Ahenda P., Vittor A.Y., Nyoka R., Gikunju S., Wachira C., Waiboci L., Umuro M., Kim A.A., Nderitu L. (2015). Seroprevalence of Infections with Dengue, Rift Valley Fever and Chikungunya Viruses in Kenya, 2007. PLoS ONE.

[94] Ofula V.O., Oundo J., Irura Z., Chepkorir E., Tigoi C., Ongus J., Coldren R., Sang R., Schoepp R., Rossi C. (2016). Evidence of presence of antibodies against selected arboviruses in Ijara and Marigat Districts, Kenya. Int. J. Infect. Dis..

[95] Oyero O.G., Ayukekbong J.A. (2014). High dengue NS1 antigenemia in febrile patients in Ibadan, Nigeria. Virus Res..

[96] Sawadogo S., Baguiya A., Yougbare F., Bicaba B.W., Nebie K., Millogo T., Kamba I., Kaba L., Sangare L., Kafando E. (2020). Seroprevalence and factors associated with IgG anti-DENV positivity in blood donors in Burkina Faso during the 2016 dengue outbreak and implications for blood supply. Transfus. Med..

[97] Schwarz N.G., Girmann M., Randriamampionona N., Bialonski A., Maus D., Krefis A.C., Njarasoa C., Rajanalison J.F., Ramandrisoa H.D., Randriarison M.L. (2012). Seroprevalence of antibodies against Chikungunya, Dengue, and Rift Valley fever viruses after febrile illness outbreak, Madagascar. Emerg. Infect. Dis..

[98] Sutherland L.J., Cash A.A., Huang Y.J., Sang R.C., Malhotra I., Moormann A.M., King C.L., Weaver S.C., King C.H., LaBeaud A.D. (2011). Serologic evidence of arboviral infections among humans in Kenya. Am. J. Trop. Med. Hyg..

[99] Tchuandom S.B., Tchouangueu T.F., Antonio-Nkondjio C., Lissom A., Djang J.O.N., Atabonkeng E.P., Kechia A., Nchinda G., Kuiate J.R. (2018). Seroprevalence of dengue virus among children presenting with febrile illness in some public health facilities in Cameroon. Pan Afr. Med. J..

[100] Vairo F., Nicastri E., Meschi S., Schepisi M.S., Paglia M.G., Bevilacqua N., Mangi S., Sciarrone M.R., Chiappini R., Mohamed J. (2012). Seroprevalence of dengue infection: A cross-sectional survey in mainland Tanzania and on Pemba Island, Zanzibar. Int. J. Infect. Dis..

[101] Ward T., Samuel M., Maoz D., Runge-Ranzinger S., Boyce R., Toledo J., Velayudhan R., Horstick O. (2017). Dengue data and surveillance in Tanzania: A systematic literature review. Trop. Med. Int. Health.

[102] Angelo K.M., Haulman N.J., Terry A.C., Leung D.T., Chen L.H., Barnett E.D., Hagmann S.H.F., Hynes N.A., Connor B.A., Anderson S. (2018). Illness among US resident student travellers after return to the USA: A GeoSentinel analysis, 2007–2017. J. Travel. Med..

[103] Badiaga S., Barrau K., Brouqui P., Durant J., Malvy D., Janbon F., Bonnet E., Bosseray A., Sotto A., Peyramont D. (2003). Imported Dengue in French University Hospitals: A 6-year survey. J. Travel. Med..

[104] Burdino E., Milia M.G., Sergi G., Gregori G., Allice T., Cazzato M.L., Lucchini A., Lipani F., Calleri G., Orofino G. (2011). Diagnosis of dengue fever in North West Italy in travelers from endemic areas: A retrospective study. J. Clin. Virol..

[105] Eldin C., Gautret P., Nougairede A., Sentis M., Ninove L., Saidani N., Million M., Brouqui P., Charrel R., Parola P. (2016). Identification of dengue type 2 virus in febrile travellers returning from Burkina Faso to France, related to an ongoing outbreak, October to November 2016. Eurosurveillance.

[106] Fang L.Q., Sun Y., Zhao G.P., Liu L.J., Jiang Z.J., Fan Z.W., Wang J.X., Ji Y., Ma M.J., Teng J. (2018). Travel-related infections in mainland China, 2014–2016: An active surveillance study. Lancet Public Health.

[107] Fourie T., Luciani L., Amrane S., Zandotti C., Leparc-Goffart I., Ninove L., Nougairede A. (2020). Dengue Virus Type 1 Infection in Traveler Returning from Benin to France, 2019. Emerg. Infect. Dis..

[108] Gautret P., Botelho-Nevers E., Charrel R.N., Parola P. (2010). Dengue virus infections in travellers returning from Benin to France, July–August 2010. Eurosurveillance.

[109] Goljan J., Myjak P., Nahorski W., Kubica-Biernat B., Felczak-Korzybska I., Kowalczyk D., Kuna A., Kotlowski A. (2010). Dengue antibodies in Polish travellers returning from the tropics. Evaluation of serological tests. Int. Marit. Health.

[110] Hashimoto T., Kutsuna S., Maeki T., Tajima S., Takaya S., Katanami Y., Yamamoto K., Takeshita N., Hayakawa K., Kato Y. (2017). A Case of Dengue Fever Imported from Burkina Faso to Japan in October 2016. Jpn. J. Infect. Dis..

[111] Jensenius M., Myrvang B. (1998). Imported fever. A diagnostic challenge. Nord. Med..

[112] Kutsuna S., Hayakawa K., Kato Y., Fujiya Y., Mawatari M., Takeshita N., Kanagawa S., Ohmagari N. (2015). Comparison of clinical characteristics and laboratory findings of malaria, dengue, and enteric fever in returning travelers: 8-year experience at a referral center in Tokyo, Japan. J. Infect. Chemother..

[113] Laferl H., Szell M., Bischof E., Wenisch C. (2006). Imported dengue fever in Austria 1990–2005. Travel. Med. Infect. Dis..

[114] Liang H., Luo L., Yang Z., Di B., Bai Z., He P., Jing Q., Zheng X. (2013). Re-Emergence of Dengue Virus Type 3 in Canton, China, 2009–2010, Associated with Multiple Introductions through Different Geographical Routes. PLoS ONE.

[115] Lim P.L., Han P., Chen L.H., MacDonald S., Pandey P., Hale D., Schlagenhauf P., Loutan L., Wilder-Smith A., Davis X.M. (2012). Expatriates ill after travel: Results from the Geosentinel Surveillance Network. BMC Infect. Dis..

[116] Moi M.L., Takasaki T., Kotaki A., Tajima S., Lim C.K., Sakamoto M., Iwagoe H., Kobayashi K., Kurane I. (2010). Importation of dengue virus type 3 to Japan from Tanzania and Cote d’Ivoire. Emerg. Infect. Dis..

[117] Nisii C., Carletti F., Castilletti C., Bordi L., Meschi S., Selleri M., Chiappini R., Travaglini D., Antonini M., Castorina S. (2010). A case of dengue type 3 virus infection imported from Africa to Italy, October 2009. Eurosurveillance.

[118] Okada K., Morita R., Egawa K., Hirai Y., Kaida A., Shirano M., Kubo H., Goto T., Yamamoto S.P. (2019). Dengue Virus Type 1 Infection in Traveler Returning from Tanzania to Japan, 2019. Emerg. Infect. Dis..

[119] Overbosch F.W., Schinkel J., Stolte I.G., Prins M., Sonder G.J.B. (2018). Dengue virus infection among long-term travelers from the Netherlands: A prospective study, 2008–2011. PLoS ONE.

[120] Suzuki T., Kutsuna S., Nakamoto T., Ota M., Ishikane M., Yamamoto K., Maeki T., Tajima S., Nakayama E., Taniguchi S. (2021). Dengue Virus Serotype 1 Exported to Japan from Cote d’Ivoire, 2019. Jpn. J. Infect. Dis..

[121] Suzuki T., Kutsuna S., Taniguchi S., Tajima S., Maeki T., Kato F., Lim C.K., Saijo M., Tsuboi M., Yamamoto K. (2017). Dengue Virus Exported from Cote d’Ivoire to Japan, June 2017. Emerg. Infect. Dis..

[122] Toro C., Trevisi P., Lopez-Quintana B., Amor A., Iglesias N., Subirats M., de Guevara C.L., Lago M., Arsuaga M., de la Calle-Prieto F. (2017). Imported Dengue Infection in a Spanish Hospital with a High Proportion of Travelers from Africa: A 9-Year Retrospective Study. Am. J. Trop. Med. Hyg..

[123] Torres-Fernandez D., Prieto Tato L.M., Perez-Ayala A., Moraleda C., Fernandez Cooke E., Blazquez-Gamero D., Rojo P., Perez Rivilla A., Epalza C. (2020). Etiology and outcome of febrile children coming from the tropics. Enferm. Infecc. Microbiol. Clin..

[124] Ujiie M., Moi M.L., Kobayashi T., Takeshita N., Kato Y., Takasaki T., Kanagawa S. (2012). Dengue virus type-3 infection in a traveler returning from Benin to Japan. J. Travel. Med..

[125] Vainio K., Noraas S., Holmberg M., Fremstad H., Wahlstrom M., Anestad G., Dudman S. (2010). Fatal and mild primary dengue virus infections imported to Norway from Africa and south-east Asia, 2008–2010. Eurosurveillance.

[126] Verschueren J., Cnops L., van Esbroeck M. (2015). Twelve years of dengue surveillance in Belgian travellers and significant increases in the number of cases in 2010 and 2013. Clin. Microbiol. Infect..

[127] Yamamoto S.P., Kasamatsu Y., Kanbayashi D., Kaida A., Shirano M., Kubo H., Goto T., Iritani N. (2019). Dengue Virus in Traveler Returning to Japan from the Democratic Republic of the Congo, 2015. Jpn. J. Infect. Dis..

[128] Mordecai E.A., Ryan S.J., Caldwell J.M., Shah M.M., LaBeaud A.D. (2020). Climate change could shift disease burden from malaria to arboviruses in Africa. Lancet Planet Health.

[129] Heath C.J., Grossi-Soyster E.N., Ndenga B.A., Mutuku F.M., Sahoo M.K., Ngugi H.N., Mbakaya J.O., Siema P., Kitron U., Zahiri N. (2020). Evidence of transovarial transmission of Chikungunya and Dengue viruses in field-caught mosquitoes in Kenya. PLOS Negl. Trop. Dis..

[130] Forsyth J.E., Mutuku F.M., Kibe L., Mwashee L., Bongo J., Egemba C., Ardoin N.M., Labeaud A.D. (2020). Source reduction with a purpose: Mosquito ecology and community perspectives offer insights for improving household mosquito management in coastal Kenya. PLOS Negl. Trop. Dis..

[131] Stoler J., Al Dashti R., Anto F., Fobil J.N., Awandare G.A. (2014). Deconstructing “malaria”: West Africa as the next front for dengue fever surveillance and control. Acta Trop..

[132] Hooft A.M., Ripp K., Ndenga B., Mutuku F., Vu D., Baltzell K., Masese L.N., Vulule J., Mukoko D., Labeaud A.D. (2017). Principles, practices and knowledge of clinicians when assessing febrile children: A qualitative study in Kenya. Malar. J..

[133] Mohammed Yusuf A., Abdurashid Ibrahim N. (2019). Knowledge, attitude and practice towards dengue fever prevention and associated factors among public health sector health-care professionals: In Dire Dawa, eastern Ethiopia. Risk Manag. Healthc. Policy.

[134] Wong P.F., Wong L.P., AbuBakar S. (2020). Diagnosis of severe dengue: Challenges, needs and opportunities. J. Infect. Public Health.

[135] Hooft A.M., Ndenga B., Mutuku F., Otuka V., Ronga C., Chebii P.K., Maina P.W., Jembe Z., Lee J., Vu D.M. (2021). High Frequency of Antibiotic Prescription in Children With Undifferentiated Febrile Illness in Kenya. Clin. Infect. Dis..

[136] Dengue: Symptoms and Treatment. https://www.cdc.gov/dengue/symptoms/index.html.

[137] Ten Bosch Q.A., Clapham H.E., Lambrechts L., Duong V., Buchy P., Althouse B.M., Lloyd A.L., Waller L.A., Morrison A.C., Kitron U. (2018). Contributions from the silent majority dominate dengue virus transmission. PLoS Pathog..

[138] Adam A., Jassoy C. (2021). Epidemiology and Laboratory Diagnostics of Dengue, Yellow Fever, Zika, and Chikungunya Virus Infections in Africa. Pathogens.

[139] Narvaez F., Gutierrez G., Perez M.A., Elizondo D., Nunez A., Balmaseda A., Harris E. (2011). Evaluation of the traditional and revised WHO classifications of Dengue disease severity. PLoS Negl. Trop. Dis..

[140] Elven J., Dahal P., Ashley E.A., Thomas N.V., Shrestha P., Stepniewska K., Crump J.A., Newton P.N., Bell D., Reyburn H. (2020). Non-malarial febrile illness: A systematic review of published aetiological studies and case reports from Africa, 1980–2015. BMC Med..

[141] Malaria. https://www.afro.who.int/health-topics/malaria.

[142] Malaria: Frequently Asked Questions (FAQs). https://www.cdc.gov/malaria/about/faqs.html.

[143] Topics: Malaria. https://www.paho.org/en/topics/malaria.

[144] Autino B., Noris A., Russo R., Castelli F. (2012). Epidemiology of malaria in endemic areas. Mediterr. J. Hematol. Infect. Dis..

[145] Malaria—Symptoms and Causes. https://www.mayoclinic.org/diseases-conditions/malaria/symptoms-causes/syc-20351184.

[146] Kazaura M. (2020). Knowledge, attitude and practices about dengue fever among adults living in Pwani Region, Tanzania in 2019. Afr. Health Sci..

[147] L’Azou M., Succo T., Kamagate M., Ouattara A., Gilbernair E., Adjogoua E., Luxemburger C. (2015). Dengue: Etiology of acute febrile illness in Abidjan, Cote d’Ivoire, in 2011–2012. Trans. R. Soc. Trop. Med. Hyg..

[148] Vu D.M., Mutai N., Heath C.J., Vulule J.M., Mutuku F.M., Ndenga B.A., Labeaud A.D. (2017). Unrecognized Dengue Virus Infections in Children, Western Kenya, 2014–2015. Emerg. Infect. Dis..

[149] Amoako N., Duodu S., Dennis F., Bonney J.H.K., Asante K., Ameh J., Mosi L., Hayashi T., Agbosu E., Pratt D. (2018). Detection of Dengue Virus among Children with Suspected Malaria, Accra, Ghana. Emerg. Infect. Dis. J..

[150] Nassar S.A., Olayiwola J.O., Bakarey A.S., Enyhowero S.O. (2019). Investigations of dengue virus and Plasmodium falciparum among febrile patients receiving care at a tertiary health facility in Osogbo, south-west Nigeria. Niger. J. Parasitol..

[151] Onyedibe K., Dawurung J., Iroezindu M., Shehu N., Okolo M., Shobowale E., Afolaranmi T., Dahal S., Maktep Y., Pama P. (2018). A cross sectional study of dengue virus infection in febrile patients presumptively diagnosed of malaria in Maiduguri and Jos plateau, Nigeria. Malawi Med. J..

[152] Stoler J., Awandare G.A. (2016). Febrile illness diagnostics and the malaria-industrial complex: A socio-environmental perspective. BMC Infect. Dis..

[153] Ndenga B.A., Mutuku F.M., Ngugi H.N., Mbakaya J.O., Aswani P., Musunzaji P.S., Vulule J., Mukoko D., Kitron U., Labeaud A.D. (2017). Characteristics of Aedes aegypti adult mosquitoes in rural and urban areas of western and coastal Kenya. PLoS ONE.

[154] Ahmad S., Dhar M., Mittal G., Bhat N.K., Shirazi N., Kalra V., Sati H.C., Gupta V. (2016). A comparative hospital-based observational study of mono- and co-infections of malaria, dengue virus and scrub typhus causing acute undifferentiated fever. Eur. J. Clin. Microbiol. Infect. Dis..

[155] Barua A., Gill N. (2016). A Comparative Study of Concurrent Dengue and Malaria Infection with their Monoinfection in a Teaching Hospital in Mumbai. J. Assoc. Physicians India.

[156] Barua A., Yeolekar M.E. (2016). Concurrent dengue and malaria coinfection: Observations from a central Mumbai hospital. Int. J. Infect. Dis..

[157] Dev N. (2019). An infection cocktail: Malaria, dengue, chikungunya and Japanese encephalitis. Trop. Doct..

[158] Gupta N., Srivastava S., Jain A., Chaturvedi U.C. (2012). Dengue in India. Indian J. Med. Res..

[159] Kaushik R.M., Varma A., Kaushik R., Gaur K.J.B.S. (2007). Concurrent dengue and malaria due to Plasmodium falciparum and *P. vivax*. Trans. R. Soc. Trop. Med. Hyg..

[160] Mandage R., Kaur C., Pramanik A., Kumar V., Kodan P., Singh A., Saha S., Pandey S., Wig N., Pandey R.M. (2020). Association of Dengue Virus and *Leptospira* Co-Infections with Malaria Severity. Emerg. Infect. Dis. J..

[161] Mushtaq M.B., Qadri M.I., Rashid A. (2013). Concurrent infection with dengue and malaria: An unusual presentation. Case Rep. Med..

[162] Rao M.R., Padhy R.N., Das M.K. (2016). Prevalence of dengue viral and malaria parasitic co-infections in an epidemic district, Angul of Odisha, India: An eco-epidemiological and cross-sectional study for the prospective aspects of public health. J. Infect. Public Health.

[163] Shah P.D., Mehta T.K. (2017). Evaluation of concurrent malaria and dengue infections among febrile patients. Indian J. Med. Microbiol..

[164] Srivatsav S., Mahalingam S., Ramineni P., Manya S. (2020). Dengue and Plasmodium falciparum Coinfection With Secondary Hemophagocytic Lymphohistiocytosis in a 3-Year-Old Boy: A Clinical Conundrum. J. Pediatr. Hematol. Oncol..

[165] Assir M.Z., Masood M.A., Ahmad H.I. (2014). Concurrent dengue and malaria infection in Lahore, Pakistan during the 2012 dengue outbreak. Int. J. Infect. Dis..

[166] Carme B., Matheus S., Donutil G., Raulin O., Nacher M., Morvan J. (2009). Concurrent dengue and malaria in Cayenne Hospital, French Guiana. Emerg. Infect. Dis..

[167] Chong S.E., Mohamad Zaini R.H., Suraiya S., Lee K.T., Lim J.A. (2017). The dangers of accepting a single diagnosis: Case report of concurrent Plasmodium knowlesi malaria and dengue infection. Malar. J..

[168] Epelboin L., Hanf M., Dussart P., Ouar-Epelboin S., Djossou F., Nacher M., Carme B. (2012). Is dengue and malaria co-infection more severe than single infections? A retrospective matched-pair study in French Guiana. Malar. J..

[169] Halsey E.S., Baldeviano G.C., Edgel K.A., Vilcarromero S., Sihuincha M., Lescano A.G. (2016). Symptoms and Immune Markers in Plasmodium/Dengue Virus Co-infection Compared with Mono-infection with Either in Peru. PLoS Negl. Trop. Dis..

[170] na Ayuthaya S.I., Wangjirapan A., Oberdorfer P. (2014). An 11-year-old boy with Plasmodium falciparum malaria and dengue co-infection. BMJ Case Rep..

[171] Khurram M., Faheem M., Umar M., Yasin A., Qayyum W., Ashraf A., Zahid Khan J., Hasnain Yasir A., Ansari Y., Asad M. (2015). Hemophagocytic Lymphohistiocytosis Complicating Dengue and Plasmodium vivax Coinfection. Case Rep. Med..

[172] Lupi O., Ridolfi F., Da Silva S., Zanini G.M., Lavigne A., Nogueira R.M.R., Cruz M.D.F.F.D., Daniel-Ribeiro C.T., Brasil P. (2016). Dengue infection as a potential trigger of an imported Plasmodium ovale malaria relapse or a long incubation period in a non-endemic malaria region. Int. J. Infect. Dis..

[173] Magalhaes B.M., Alexandre M.A., Siqueira A.M., Melo G.C., Gimaque J.B., Bastos M.S., Figueiredo R.M., Carvalho R.C., Tavares M.A., Naveca F.G. (2012). Clinical profile of concurrent dengue fever and Plasmodium vivax malaria in the Brazilian Amazon: Case series of 11 hospitalized patients. Am. J. Trop. Med. Hyg..

[174] Magalhaes B.M., Siqueira A.M., Alexandre M.A., Souza M.S., Gimaque J.B., Bastos M.S., Figueiredo R.M., Melo G.C., Lacerda M.V., Mourao M.P. (2014). *P. vivax* malaria and dengue fever co-infection: A cross-sectional study in the Brazilian Amazon. PLoS Negl. Trop. Dis..

[175] Mendonca V.R., Andrade B.B., Souza L.C., Magalhaes B.M., Mourao M.P., Lacerda M.V., Barral-Netto M. (2015). Unravelling the patterns of host immune responses in Plasmodium vivax malaria and dengue co-infection. Malar. J..

[176] Santana Vdos S., Lavezzo L.C., Mondini A., Terzian A.C., Bronzoni R.V., Rossit A.R., Machado R.L., Rahal P., Nogueira M.C., Nogueira M.L. (2010). Concurrent Dengue and malaria in the Amazon region. Rev. Soc. Bras. Med. Trop..

[177] Selvaretnam A.A.P., Sahu P.S., Sahu M., Ambu S. (2016). A review of concurrent infections of malaria and dengue in Asia. Asian Pac. J. Trop. Biomed..

[178] Serre N., Franco L., Sulleiro E., Rubio J.M., Zarzuela F., Molero F., Tenorio A. (2015). Concurrent Infection With Dengue Type 4 and Plasmodium falciparum Acquired in Haiti. J. Travel. Med..

[179] Shams N., Amjad S., Yousaf N., Ahmed W., Seetlani N.K., Qaisar N., Samina (2016). Predictors of Severity of Dengue Fever in Tertiary Care Hospitals. J. Liaquat Univ. Med. Health Sci..

[180] Thaha M., Pranawa, Yogiantoro M., Tanimoto M., Tomino Y. (2008). Acute renal failure in a patient with severe malaria and dengue shock syndrome. Clin. Nephrol..

[181] Ward D.I. (2006). A case of fatal Plasmodium falciparum malaria complicated by acute dengue fever in East Timor. Am. J. Trop. Med. Hyg..

[182] Zhao Y., Wu X., Liao F. (2018). Severe Cerebral Falciparum Malaria with Dengue Coinfection: A Case Report. Iran. J. Parasitol..

[183] Baba M., Logue C.H., Oderinde B., Abdulmaleek H., Williams J., Lewis J., Laws T.R., Hewson R., Marcello A., Agaro P.D. (2013). Evidence of arbovirus co-infection in suspected febrile malaria and typhoid patients in Nigeria. J. Infect. Dev. Ctries..

[184] Galani B.R.T., Mapouokam D.W., Simo F.B.N., Mohamadou H., Chuisseu P.D.D., Njintang N.Y., Moundipa P.F. (2021). Investigation of dengue–malaria coinfection among febrile patients consulting at Ngaoundere Regional Hospital, Cameroon. J. Med. Virol..

[185] Kolawole O.M., Seriki A.A., Irekeola A.A., Bello K.E., Adeyemi O.O. (2017). Dengue virus and malaria concurrent infection among febrile subjects within Ilorin metropolis, Nigeria. J. Med. Virol..

[186] Liu E., Vu D., Boothroyd D., Ndenga B., Onyango W., Okuta V., Labeaud A.D. (2016). Evaluation of the Health-Related Quality of Life of Children with Dengue and Malaria in Western Kenya. Open Forum Infectious Diseases.

[187] Monamele G.C., Demanou M. (2018). First documented evidence of dengue and malaria co-infection in children attending two health centers in Yaounde, Cameroon. Pan Afr. Med. J..

[188] Olufisayo A.A., Johnson A.A. (2016). Incidence of dengue virus infections in febrile episodes in Ile-Ife, Nigeria. Afr. J. Infect. Dis..

[189] Raut C.G., Rao N.M., Sinha D.P., Hanumaiah H., Manjunatha M.J. (2015). Chikungunya, dengue, and malaria co-infection after travel to Nigeria, India. Emerg. Infect. Dis..

[190] Sow A., Loucoubar C., Diallo D., Faye O., Ndiaye Y., Senghor C.S., Dia A.T., Faye O., Weaver S.C., Diallo M. (2016). Concurrent malaria and arbovirus infections in Kedougou, southeastern Senegal. Malar. J..

[191] Stoler J., Delimini R.K., Bonney J.H., Oduro A.R., Owusu-Agyei S., Fobil J.N., Awandare G.A. (2015). Evidence of recent dengue exposure among malaria parasite-positive children in three urban centers in Ghana. Am. J. Trop. Med. Hyg..

[192] Vu D.M., Ripp K., Mutai N., Ndenga B.A., Heath C., Labeaud A.D. (2016). Dengue virus and malaria co-infection in Kenyan children. Ann. Glob. Health.

[193] Kotepui M., Kotepui K.U., Milanez G.J., Masangkay F.R. (2020). Prevalence of and risk factors for severe malaria caused by Plasmodium and dengue virus co-infection: A systematic review and meta-analysis. Infect. Dis. Poverty.

[194] Kotepui M., Kotepui K.U. (2019). Prevalence and laboratory analysis of malaria and dengue co-infection: A systematic review and meta-analysis. BMC Public Health.

[195] Carabali M., Hernandez L.M., Arauz M.J., Villar L.A., Ridde V. (2015). Why are people with dengue dying? A scoping review of determinants for dengue mortality. BMC Infect. Dis..

[196] Toledo J., George L., Martinez E., Lazaro A., Han W.W., Coelho G.E., Runge Ranzinger S., Horstick O. (2016). Relevance of Non-communicable Comorbidities for the Development of the Severe Forms of Dengue: A Systematic Literature Review. PLoS Negl. Trop. Dis..

[197] Wilder-Smith A., Leong W.Y. (2019). Risk of severe dengue is higher in patients with sickle cell disease: A scoping review. J. Travel Med..

[198] Leslie T.E. (2011). Dengue Fever and the Quandary of Race. Lat. Am. Caribb. Ethn. Stud..

[199] Fujimura J.H., Rajagopalan R. (2011). Different differences: The use of ‘genetic ancestry’ versus race in biomedical human genetic research. Soc. Stud. Sci..

[200] McAfee S. (2017). Race is a Social Construct.

[201] Borrell L.N., Elhawary J.R., Fuentes-Afflick E., Witonsky J., Bhakta N., Wu A.H.B., Bibbins-Domingo K., Rodriguez-Santana J.R., Lenoir M.A., Gavin J.R. (2021). Race and Genetic Ancestry in Medicine—A Time for Reckoning with Racism. N. Engl. J. Med..

[202] Malaria—Biology. https://www.cdc.gov/malaria/about/biology/index.html.

[203] Xavier-Carvalho C., Cardoso C.C., de Souza Kehdy F., Pacheco A.G., Moraes M.O. (2017). Host genetics and dengue fever. Infect. Genet. Evol..

[204] Coffey L.L., Mertens E., Brehin A.C., Fernandez-Garcia M.D., Amara A., Despres P., Sakuntabhai A. (2009). Human genetic determinants of dengue virus susceptibility. Microbes Infect..

[205] Stephens H.A. (2010). HLA and other gene associations with dengue disease severity. Curr. Top. Microbiol. Immunol..

[206] Gupta S., Agarwal A., Kumar A., Biswas D. (2018). Genome-Wide Analysis to Identify HLA Factors Potentially Associated With Severe Dengue. Front. Immunol..

[207] Xavier Eurico De Alencar L., De Mendonça Braga-Neto U., José Moura Do Nascimento E., Tenório Cordeiro M., Maria Silva A., Alexandre Antunes De Brito C., Da Silva M.D.P.C., Gil L.H.V.G., Montenegro S.M.L., Marques E.T.D.A. (2013). HLA-B*44 Is Associated with Dengue Severity Caused by DENV-3 in a Brazilian Population. J. Trop. Med..

[208] Murugananthan K., Subramaniyam S., Kumanan T., Owens L., Ketheesan N., Noordeen F. (2018). Blood group AB is associated with severe forms of dengue virus infection. VirusDisease.

[209] Pare G., Neupane B., Eskandarian S., Harris E., Halstead S., Gresh L., Kuan G., Balmaseda A., Villar L., Rojas E. (2020). Genetic risk for dengue hemorrhagic fever and dengue fever in multiple ancestries. EBioMedicine.

[210] Sierra B., Triska P., Soares P., Garcia G., Perez A.B., Aguirre E., Oliveira M., Cavadas B., Regnault B., Alvarez M. (2017). OSBPL10, RXRA and lipid metabolism confer African-ancestry protection against dengue haemorrhagic fever in admixed Cubans. PLoS Pathog..

[211] Bravo J.R., Guzman M.G., Kouri G.P. (1987). Why dengue haemorrhagic fever in Cuba? 1. Individual risk factors for dengue haemorrhagic fever/dengue shock syndrome (DHF/DSS). Trans. R. Soc. Trop. Med. Hyg..

[212] Sierra B.D.L.C., Garcia G., Perez A.B., Morier L., Alvarez M., Kouri G., Guzman M.G. (2006). Ethnicity and difference in dengue virus-specific memory T cell responses in Cuban individuals. Viral. Immunol..

[213] Sierra B.D.L.C., Kouri G., Guzman M.G. (2007). Race: A risk factor for dengue hemorrhagic fever. Arch. Virol..

[214] Oliveira M., Saraiva D.P., Cavadas B., Fernandes V., Pedro N., Casademont I., Koeth F., Alshamali F., Harich N., Cherni L. (2018). Population genetics-informed meta-analysis in seven genes associated with risk to dengue fever disease. Infect. Genet. Evol..

[215] Halstead S.B., Streit T.G., Lafontant J.G., Putvatana R., Russell K., Sun W., Kanesa-Thasan N., Hayes C.G., Watts D.M. (2001). Haiti: Absence of dengue hemorrhagic fever despite hyperendemic dengue virus transmission. Am. J. Trop. Med. Hyg..

[216] Chacón-Duque J.C., Adhikari K., Avendaño E., Campo O., Ramirez R., Rojas W., Ruiz-Linares A., Restrepo B.N., Bedoya G. (2014). African genetic ancestry is associated with a protective effect on Dengue severity in colombian populations. Infect. Genet. Evol..

[217] Boillat-Blanco N., Klaassen B., Mbarack Z., Samaka J., Mlaganile T., Masimba J., Franco Narvaez L., Mamin A., Genton B., Kaiser L. (2018). Dengue fever in Dar es Salaam, Tanzania: Clinical features and outcome in populations of black and non-black racial category. BMC Infect. Dis..

[218] Blanton R.E., Silva L.K., Morato V.G., Parrado A.R., Dias J.P., Melo P.R., Reis E.A., Goddard K.A., Nunes M.R., Rodrigues S.G. (2008). Genetic ancestry and income are associated with dengue hemorrhagic fever in a highly admixed population. Eur. J. Hum. Genet..

[219] Mawson A.R. (2013). Retinoids, race and the pathogenesis of dengue hemorrhagic fever. Med. Hypotheses.

[220] Restrepo B.N., Arboleda M., Ramirez R., Alvarez G. (2011). Serum platelet-activating factor acetylhydrolase activity in dengue patients of African or mestizo descendency. Biomedica.

[221] Rojas Palacios J.H., Alzate A., Martinez Romero H.J., Concha-Eastman A.I. (2016). AfroColombian ethnicity, a paradoxical protective factor against Dengue. Colomb. Med..

[222] Malik A., Earhart K., Mohareb E., Saad M., Saeed M., Ageep A., Soliman A. (2011). Dengue hemorrhagic fever outbreak in children in Port Sudan. J. Infect. Public Health.

[223] The Hidden Burden of Dengue Fever in West Africa. https://www.infectioncontroltoday.com/view/hidden-burden-dengue-fever-west-africa.

